# Does SARS-CoV-2 Infection Increase Risk of Neuropsychiatric and Related Conditions? Findings from Difference-in-Differences Analyses

**DOI:** 10.21203/rs.3.rs-5621095/v1

**Published:** 2025-01-08

**Authors:** Yong Chen, Yiwen Lu, Jiayi Tong, Dazheng Zhang, Jiajie Chen, Lu Li, Yuqing Lei, Ting Zhou, Leyna Aragon, Michael Becich, Saul Blecker, Nathan Blum, Dimitri Christakis, Mady Hornig, Maxwell Hornig-Rohan, Ravi Jhaveri, William Jones, Amber Keebler, Kelly Kelleher, Susan Kim, Abu Mosa, Kathleen Pajer, Jonathan Platt, Hayden Schwenk, Bradley Taylor, Levon Utidjian, David Williams, Raghuram Prasad, Josephine Elia, Christopher Forrest

**Affiliations:** University of Pennsylvania; University of Pennsylvania; Johns Hopkins Bloomberg School of Public Health; University of Pennsylvania; University of Pennsylvania; University of Pennsylvania; University of Pennsylvania; University of Pennsylvania; University of New Mexico; University of Pittsburgh School of Medicine; Department of Population Health, New York University Grossman School of Medicine; Division of Developmental and Behavioral Pediatrics, Children’s Hospital of Philadelphia; Seattle Children’s Research Institute; RECOVER Patient, Caregiver, or Community Advocate Representative; Division of Infectious Diseases, Ann & Robert H. Lurie Children’s Hospital of Chicago; Info Duke Clinical Research Institute, Durham, United States; University of Nebraska Medical Center; The Abigail Wexner Research Institute at Nationwide Children’s Hospital; University of California, San Francisco; University of Missouri School of Medicine; University of Ottawa Department of Psychiatry, Children’s Hospital of Eastern Ontario; Department of Epidemiology, The University of Iowa College of Public Health; Stanford School of Medicine; Medical College of Wisconsin; Applied Clinical Research Center, Children’s Hospital of Philadelphia; University of Michigan Medical School; Department of Child and Adolescent Psychiatry, Children’s Hospital of Philadelphia, Perelman School of Medicine, the University of Pennsylvania; Department of Pediatrics, Nemours Children’s Health Delaware, Sydney Kimmel School of Medicine; Children’s Hospital of Philadelphia

## Abstract

The COVID-19 pandemic has been associated with increased neuropsychiatric conditions in children and youths, with evidence suggesting that SARS-CoV-2 infection may contribute additional risks beyond pandemic stressors. This study aimed to assess the full spectrum of neuropsychiatric conditions in COVID-19 positive children (ages 5–12) and youths (ages 12–20) compared to a matched COVID-19 negative cohort, accounting for factors influencing infection risk. Using EHR data from 25 institutions in the RECOVER program, we conducted a retrospective analysis of 326,074 COVID-19 positive and 887,314 negative participants matched for risk factors and stratified by age. Neuropsychiatric outcomes were examined 28 to 179 days post-infection or negative test between March 2020 and December 2022. SARS-CoV-2 positivity was confirmed via PCR, serology, or antigen tests, while negativity required negative test results and no related diagnoses. Risk differences revealed higher frequencies of neuropsychiatric conditions in the COVID-19 positive cohort. Children faced increased risks for anxiety, OCD, ADHD, autism, and other conditions, while youths exhibited elevated risks for anxiety, suicidality, depression, and related symptoms. These findings highlight SARS-CoV-2 infection as a potential contributor to neuropsychiatric risks, emphasizing the importance of research into tailored treatments and preventive strategies for affected individuals.

## Introduction

Increased neuropsychiatric sequelae associated with the COVID-19 pandemic has been reported worldwide.^1,2^ However, there remains uncertainty whether these can be directly attributed to SARS-CoV-2 infection or the broader pandemic stressors and mitigation strategies.^2–4^ Similar to adults, children and youths are also susceptible to experiencing enduring neuropsychiatric and related conditions after an acute COVID-19 infection.^5,6^ Although significant research has been conducted on PASC in the adult population, there remains a notable gap in studies pertaining to pediatric cases.^7–9^ Children and youths often exhibit distinct symptoms compared to adults and typically experience a milder acute disease trajectory, with a reduced risk of hospitalization or mortality, especially in cases where pre-existing conditions are absent.^10,11^ Given these variations in acute infection profiles and prevalence in children and youths as compared with adults, it is imperative to separately investigate the characteristics of PASC in the pediatric population in well-controlled studies.

There are existing studies with large pediatric samples investigating neuropsychiatric conditions in pediatric populations with and without COVID-19 infection.^12–15^ However, the results remain inconclusive due to limitations such as the reliance solely on diagnoses to identify COVID-19 positive and negative cohorts, with only a subset being confirmed with testing.^13,14^ Given that COVID-19 symptoms are often mild or absent in children, some infected individuals may have been misclassified.^12–14^ These studies likely underestimated the prevalence of mental health conditions, as many DSM-5-based diagnoses used by clinicians cannot be fully matched to ICD-10-CM codes.^12–15^

In our study, the large EHR data set allowed COVID-19 negative cohorts of sufficient size matched for risk factors and stratified by age.^15^ We used both diagnosis and PCR, antigen, or serology tests to reliably identify COVID-19 positive and negative groups. Neuropsychiatric and related conditions were identified by a typology developed to query EHR data for the full spectrum of DSM-5 disorders.^16^ The primary objective of this retrospective cohort study was to ascertain the risk of developing neuropsychiatric and related conditions after the pandemic in children and youths who had tested positive for COVID-19 compared to those who tested negative and never had a positive test at the same time interval. To achieve this, we utilized EHR data collected from twenty-five children’s hospitals and healthcare institutions across the United States from the RECOVER program. Initially, we calculated the raw frequency of any neuropsychiatric and related conditions, both before and after the onset of the pandemic. Subsequently, we conducted an interrupted time series analysis to determine whether contracting SARS-CoV-2 increased the risk of being diagnosed with neuropsychiatric and related conditions, compared to the SARS-19 negative group, both groups being exposed to the pandemic psychosocial stressors.

## Results

### Frequency of Post-acute Neuropsychiatric Related Events for COVID-19-positive and COVID-19-negative patients

As shown in Tables 2 and 3, there were small increases in frequency of any neuropsychiatric and related condition in the post-COVID phase (compared to pre-COVID) for both COVID-19 positive and COVID-19 negative groups in the children (COVID 19 positive cohort:12·45% to 14·01%; COVID 19-negative cohort: 11·6% to 12·48%) as well as for youths (COVID-19 positive cohort: 16·0% to 17·86%; COVID 19 negative cohorts: 15·55% to 16·76%).

During the post-acute phase, both the child and youth COVID-19 positive groups displayed a higher frequency than their respective COVID-19 negative groups in the composite outcome and across various categories, including adverse childhood experience, anxiety disorders, mood disorders, neurocognitive disorders, neurodevelopmental disorders, sleep-wake disorders, standalone symptoms, and substance use and dependence. Additionally, the child COVID-19 positive group has a higher prevalence than the COVID-19 negative group in eating and feeding disorders, intentional self-harm/suicidality, personality disorders, psychotic disorders, and tic disorders.

### Risk Difference of Post-acute Neuropsychiatric Outcomes after SARS-CoV-2 Infection

As shown in Figs. 2 and 3, after propensity score matching and interrupted time analysis, both the children and youths COVID-19 positive groups retained significant risk differences compared to their respective negative groups in the composite outcome (children: 0·96%, 95% CI [0·75%, 1.16%]; the youth: 0·84%, [0·53%, 1.15%]). The children COVID-19 positive group also exhibited significant risk differences for anxiety disorder (0s26%, [0·19%, 0·33%]), OCD (0·02%, [0·00%, 0·04%]), somatoform disorder (0·03%, [0·00%, 0·05%]), stress disorder (0·08%, [0·02%, 0·14%]), avoidant/restrictive food intake (0·07%, [0·03%, 0·11%]), bipolar disorder (0·01%, [0·00%, 0·02%]), delirium (0·04%, [0·02%, 0·06%]), ADHD (0·11%, [0·02%, 0·21%]), autism spectrum disorder (0·10%, [0·02%, 0·18%]), communication/motor disorder (0·38%, [0·25%, 0·52%]), and intellectual disability (0·12%, [0·05%, 0·20%]), and tic disorder (0·05%, [0·02%, 0·08%]).

For the youth cohorts, the COVID-19 positive group had significantly higher risk difference compared to the COVID-19 negative cohort in anxiety disorder (0·26%, [0·05%, 0·48%]), suicidality (0·11%, [0·02%, 0·19%]), minor depression (0·21%, [0·05%, 0·37%]), delirium (0·08%, [0·03%, 0·14%]), ADHD (0·33% [0·16%, 0·50%]), intellectual disability (0·09%, [0·01%, 0·17%]), insomnia (0·13%, [0·06%, 0·21%]), and anxiety standalone symptoms (0·05%, [0·00%, 0·10%]), attention standalone symptoms (0·08%, [0·03%, 0·14%]), depressive standalone symptoms (0·02%, [0·00%, 0·04%]).

Selective psychotropic medications with the potential to decrease susceptibility to SARS-CoV-2 infection were used by 0·68% of COVID-19 positive children and 0·75% of negative children aged 5–12 years. Among youths, these medications were used by 5·09% of COVID-19 positive patients and 5·36% of negative patients. Detailed results can be found in Supplementary Materials Section 3.

## Discussion

Infections have long been linked to neuropsychiatric disorders, as evidenced by reports from the 1890 influenza epidemic, the 1918 Spanish flu, and more recently, a Danish nationwide study.^17^ This study found that children and adolescents who were hospitalized for infections faced an increased risk of subsequent diagnoses of neuropsychiatric disorders and higher rates of psychotropic medication prescriptions. The highest risks following infections were associated with conditions such as schizophrenia, OCD, personality and behavioral disorders, intellectual disability, autism, ADHD, ODD, conduct disorders, and tic disorders.^17^ In this study, the primary objective was to investigate the impact of COVID-19 infection on the potential risk of post-acute sequelae neuropsychiatric and related conditions for both children and youths. Using the real-world EHR data from twenty-five health institutions in the RECOVER program, we conducted the retrospective cohort study of patients 5 to 20 years of age with documented SARS-CoV-2 infection compared to those with a negative test. Our findings, which demonstrate increased rates of neuropsychiatric and related conditions in both COVID-19 positive and negative cohorts during the post-COVID phase, align with global reports highlighting the combined effects of SARS-CoV-2 infection and broader pandemic stressors.^18^ Similarly, the higher frequency rates observed in older age groups in both COVID-19 positive and negative cohorts (1·56% and 0·88%, respectively, for ages 5–11, and 1·86% and 1·21%, respectively, for ages 12–20) echo prior studies suggesting that adolescents and young adults may be disproportionately affected by both the viral infection and pandemic stress compared to younger children.^18^ Recent large-scale studies using EHR data further support this, reporting a higher likelihood of developing new mental health disorders in both COVID-19 positive and negative adolescents compared to younger children.^14^

The key findings from our study show that both children and youth in the COVID-19 positive groups retained significant risk differences compared to their respective negative groups for the composite neuropsychiatric outcome (as shown in Table 3 and Fig. 2). The risk difference was slightly higher in children than in youths. Additionally, differences across diagnostic categories were observed between the two age groups. Among children with infection, the highest risk difference was seen for communication/motor disorders, followed by anxiety, intellectual disability, ADHD, and autism spectrum disorder. Other conditions, such as stress-related disorders, avoidant/restrictive food intake, tics, delirium, somatoform disorders, OCD, and bipolar disorder, had risk differences ranging from 0·08% to 0·01%. In youth with infection, the highest significant risk difference was for anxiety disorders, followed by minor depression, standalone attention symptoms, insomnia, and suicidality. Intellectual disability and standalone symptoms of anxiety and depression had risk differences ranging from 0·09% to 0·02%. The small increases in risk found in our study support studies indicating that infections may account for only a small proportion of the risk for mental disorders.^19^ That same study also showed that polygenic risk scores for infections were associated with modest increase in risk for ADHD, major depression, and schizophrenia. In our study, increased risk for ADHD and minor depression were found in the COVID-19 positive child and youth cohorts respectively while risks for disorders that are more common in the older age ranges would be less likely to be detected.

Our study has several notable strengths. Firstly, by leveraging EHR data from over twenty clinical institutions nationwide as part of the RECOVER program, our research presents the most comprehensive investigation on U.S. children and youths to date, exploring the impact of SARS-CoV-2 infection on the neuropsychiatric and related conditions. Secondly, our approach included a more extended follow-up period than most existing studies. Specifically, our follow-up extended until December 2022, encompassing the period that included the emergence of the Omicron variant. Thirdly, we accounted for pre-infection differences in neuropsychiatric and related condition risks by employing the difference-in-differences method. This approach allowed us to examine the effects directly attributable to SARS-CoV-2 infection while controlling for any baseline disparities in neuropsychiatric and related conditions. Additionally, we enhanced our analysis by adjusting for over 200 potential confounders through propensity score stratification. This method ensured a balanced comparison between the SARS-CoV-2-infected and non-infected groups. Lastly, our study’s comprehensive scope, examining 50 neuropsychiatric and related outcomes at both individual disorder and category levels, facilitated a comprehensive exploration of the patterns and impacts of SARS-CoV-2 infection on neuropsychiatric and related conditions, whereby studies using limited ICD codes for anxiety and depression did not detect a pandemic effect.^20^ This approach offers a better understanding of the association and effects of various factors on neuropsychiatric dysfunction in the context of the pandemic.

Our study is subject to several limitations that can be considered for future studies. Firstly, identifying a high-quality COVID-19 negative group presents a significant challenge. To mitigate potential misclassification of negative status, we have utilized multiple tests, including PCR, antigen, and serology test results, in addition to diagnosis codes for COVID-19 and long COVID, to refine our definition of the COVID-19 negative group. Despite these efforts, the rapid and dynamic developmental changes experienced by children and youths, such as the physical growth and changes in physiological, cognitive, emotional, and social domains, suggest that further enhancements in control selection methods could improve the reliability of our findings. Secondly, although we implemented rigorous methods to ensure comprehensive data collection, certain biases may be intrinsic to our study. For example, in youths with more severe symptoms, parents may have been more likely to disclose additional health-related information, potentially leading to reporting biases. Differential access to clinicians with the appropriate expertise to evaluate neuropsychiatric issues could also have contributed to the underascertainment of such conditions. Thirdly, while our analysis incorporated an extensive list of potential confounders available within the EHR database, the inherent limitations of EHR data completeness may still introduce potential confounding bias. Moreover, our analysis did not account for participants who may have been infected several times during the study period, a factor that could become increasingly relevant in the later stages of the pandemic.

In summary, in both COVID positive and negative cohorts, we found small increases in frequency in composite neuropsychiatric and related outcomes, slightly higher in the COVID positive group and in the older age groups. These small increases are similar to those reported in other studies and attributed to the combined COVID-19 viral infection and broad pandemic stressors.^18,21^

While the frequency attributed to the combined viral infection and pandemic stress, and the risk attributed to the viral infection may be small, these raise concern in a pediatric population given that childhood conditions often have lifelong consequences.^22,23^

Our results, therefore, indicate an urgent need for well-controlled studies that investigate not only COVID-19 but other infections, known to affect the CNS. Pediatric studies also require cohorts with narrower age stratification, cohorts that also include the prenatal period, and adequate follow-up to control for the rapid neurodevelopmental changes.

### Role of the funding source

The funders of the study had no role in study design, data collection, data analysis, data interpretation, or writing of the manuscript.

## Methods

### Study design and participants

We conducted a retrospective cohort study using the pediatric EHR cohort of the NIH Researching COVID to Enhance Recovery (RECOVER) Initiative, which seeks to understand, treat, and prevent long COVID (more information on RECOVER https://recoverCOVID.org/). The pediatric RECOVER EHR network spans 38 health systems across the United States, of which 25 were included in the study. The Institutional Review Board (IRB) obtained approval under Biomedical Research Alliance of New York (BRANY) protocol #21–08-508, with a waiver of consent and HIPAA authorization. The participating institutions in this study include Ann & Robert H. Lurie Children’s Hospital of Chicago, Children’s Hospital Colorado, Children’s Hospital of Philadelphia, Children’s National Medical Center, Cincinnati Children’s Hospital Medical Center, Duke University, Medical College of Wisconsin, Medical University of South Carolina (MUSC), Montefiore, Nationwide Children’s Hospital, Nemours Children’s Health System (inclusive of the Delaware and Florida health system), New York University School of Medicine, Northwestern University, OCHIN, Seattle Children’s Hospital, Stanford Children’s Health, University of California, San Francisco, University of Iowa Healthcare, University of Michigan, University of Missouri, University of Nebraska Medical Center, University of Pittsburgh, Vanderbilt University Medical Center, Wake Forest Baptist Health, and Weill Cornell Medical College. Detailed data description can be found in Supplementary Materials Section 1.

In the construction of our COVID-19 positive cohort, we began by identifying individuals who received their first positive COVID-19 PCR, antigen, or serology test and a diagnosis of COVID-19/PASC within the study period from March 1st, 2020, to December 3rd, 2022 (N = 1,017,542). From this initial group, we subsequently filtered for those with at least one medical visit occurring between 28 and 179 days after the index date (follow-up interval)^24–27^ (N = 787,370) and at least one visit within the 7 days to 24 months leading up to the index date (baseline interval) (N = 676,582). We included only the patients with complete variable records (n = 488,606), and we refined the positive cohort with age constraints between five and twenty when the study period starts and complete records (N = 326,074). Among these individuals, we identified a child cohort with ages 5–11 years (N = 141,349) and a youth cohort with ages 12–20 (N = 184,725).

**Figure 1:**
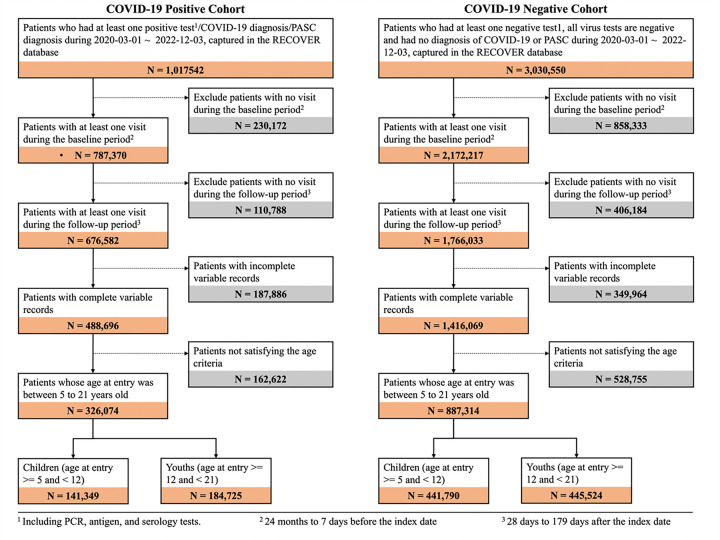
Selection of participants for both COVID-19-positive and COVID-19-negative patients, stratified by age (children and youths).

**Figure 2:**
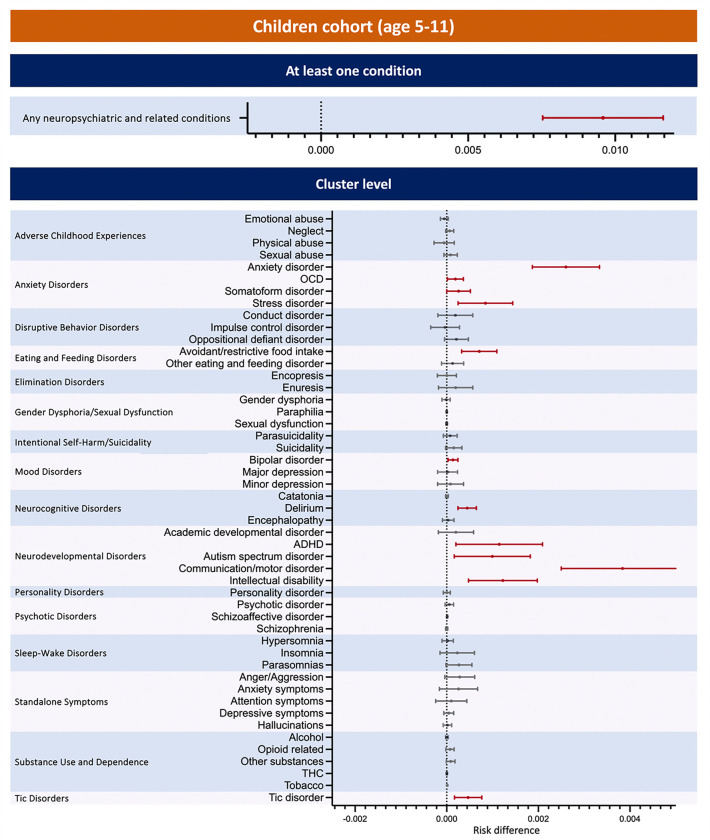
Risk Difference of post-acute COVID-19 neuropsychiatric and related conditions compared with the COVID-19-negative cohort in children (age 5~11). Outcomes consisted of multiple cluster level conditions in adverse childhood experiences, anxiety disorders, disruptive behavior disorders, eating and feeding disorders, elimination disorders, gender dysphoria/sexual dysfunction, intentional self-harm/suicidality, mood disorders, neurocognitive disorders, neurodevelopmental disorders, personality disorders, psychotic disorders, sleep-wake disorders, standalone symptoms, substance use and dependence, and tic disorders. The composite outcome (any neuropsychiatric and related conditions) refers to occurrence of any neuropsychiatric and related outcome listed.

**Figure 3:**
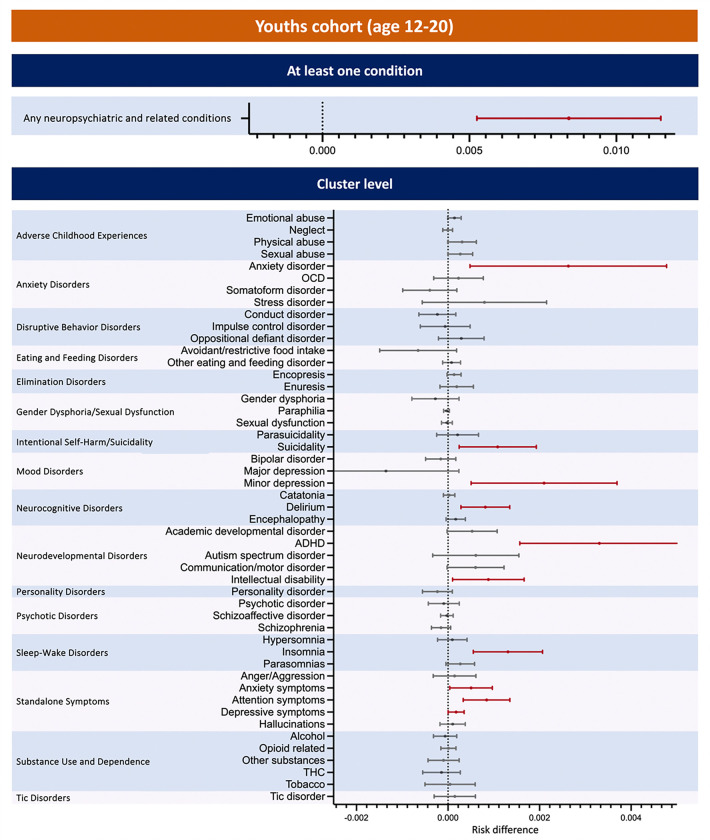
Risk Difference of post-acute COVID-19 neuropsychiatric and related conditions compared with the COVID-19-negative cohort in youths (age 12~20). Outcomes consisted of multiple cluster level conditions in adverse childhood experiences, anxiety disorders, disruptive behavior disorders, eating and feeding disorders, elimination disorders, gender dysphoria/sexual dysfunction, intentional self-harm/suicidality, mood disorders, neurocognitive disorders, neurodevelopmental disorders, personality disorders, psychotic disorders, sleep-wake disorders, standalone symptoms, substance use and dependence, and tic disorders. The composite outcome (any neuropsychiatric and related conditions) refers to occurrence of any neuropsychiatric and related outcome listed.

We then constructed a COVID-19 negative group composed of individuals who were not part of the COVID-19 positive cohort, had at least one negative COVID-19 PCR, antigen, or serology test within the same study period, and no diagnoses of COVID-19 or PASC (N = 3,030,550). For this COVID-19 negative group, we imputed index dates randomly from the distribution of index dates observed in the COVID-19 cohort, ensuring that both cohorts shared a similar distribution of follow-up times. We further required that patients in the COVID-19 negative cohort must have had at least one visit between 28 and 179 days after the imputed index date as the follow up period (N = 2,172,217) and at least one visit occurring between 7 days to 24 months before the imputed index date as the baseline period (N = 1,766,033). Similar to the COVID-19 positive cohort, we only included patients with complete variable records (N = 1,416,069) and satisfying age constraints between five and twenty at the start of the study period (N = 887,314). We further stratified the children cohort with ages from five to eleven (N = 441,790) and the youth cohort with ages from twelve to twenty (N = 445,524). Figure 1 displays attrition tables for both COVID-19 positive and negative cohorts.

In this research, we utilized covariates assessed before the index date. The predefined covariates were determined based on prior knowledge.^28,29^ The predefined covariates included age, race (Asian/PI, black/AA, Hispanic, white, multiple, and other), gender (male, female, and other), hospital, body mass index, and hospital utilization including number of ED visits, number of inpatient and outpatient encounters, PMCA index, number of negative tests prior to the entry of cohorts, and medical history. The baseline description of covariates in both cohorts is presented in Table 1.

We also evaluated the use of selective psychotropic medications, reported to be activators of Sigma 1-receptor ligand, of varying affinity, as some prior data suggested their potential capacity to decrease susceptibility to SARS-CoV-2 infection. These included SSRIs (fluvoxamine, fluoxetine, citalopram, and escitalopram) and antipsychotics (haloperidol, chlorpromazine, and fluphenazine).^30,31^ We evaluated the prevalence of usage of the above medications in both COVID-19 positive patients and the negative cohort to ensure that SSRI usage did not introduce imbalance or bias into our study results.

### Outcomes

The outcomes were predetermined based on our prior research on systematically characterizing the post-acute effects of SARS-CoV-2 infection.^32^ We specify our outcomes based on Systematized Nomenclature of Medicine (SNOMED),^33^ and a typology developed to query aggregated, standardized EHR data for the full spectrum of neuropsychiatric and related conditions. This typology included the pediatric DSM-5 disorder categories including anxiety, OCD, somatic, stress, disruptive behavior, feeding and eating, elimination, gender dysphoria/sexual dysfunction, mood, neurocognitive, neurodevelopmental, personality, psychotic, sleep-wake, substance use, and dependence disorders.^34^ Expansion beyond DSM-5 disorders included intentional self-harm, catatonia, encephalopathies, standalone symptoms, tic disorders, and adverse childhood experiences.^16^

We also specified a composite outcome of any neuropsychiatric and related condition. Supp Table 1 in Supplementary Materials Section 2 details the definition of the outcomes. Frequencies of each outcome were assessed 24 months to 7 days before and 28 days to 179 days after the index date for children and youths, respectively (Table 2, 3).

### Statistical Analyses

We defined the pre-COVID period as the span from 24 months to 7 days before the index date and the post-COVID period as the period from 28 to 179 days after the index date (the post-acute phase). For each neuropsychiatric and related condition, we calculated its frequency by dividing the number of patients who were diagnosed during each of the defined periods.

To assess differences in the risk of neuropsychiatric and related conditions between COVID-19 positive and negative patients, we conducted an interrupted time-series analysis using a two-sample proportion test with stratified cohorts of children and youths. To mitigate the potential impact of measured confounding factors, we employed a propensity score matching method with the covariates outlined in the Covariates section. After matching, we assessed the standardized mean difference (SMD) for each covariate, employing a cutoff value of 0·1. Subsequently, we compared the risk difference in neuropsychiatric and related conditions between the COVID-19 positive and the COVID-19 negative cohort. The characteristic balance results before and after propensity score matching are presented in Supplementary Materials Section 4.

### Sensitivity Analysis

We performed comprehensive sensitivity analyses to assess the robustness of our findings. Initially, we conducted an analysis without age stratification and documented the results in Section 5 of the Supplementary Materials. We also performed an analysis with a different control group, which was defined as patients with at least one negative test and one non-COVID respiratory disease diagnosis within 30 days of the negative test. Details of the study design and results are documented in Section 6 of the Supplementary Materials. Furthermore, our sensitivity analysis included subgroup analyses in Sections 7–12 of the Supplementary Materials based on gender (male and female), race/ethnicity (Asian/Pacific Islander (PI), Black/African-American(AA), Hispanic, and White), obesity, hospitalization status (non-hospitalized, hospitalized, and admitted to ICU), severity of symptoms (asymptomatic, mild, moderate, and severe), and time frames corresponding to predominant virus variants (pre-Delta, Delta, and Omicron).

## Figures and Tables

**Table 1:** Baseline demographic and health characteristics of COVID-19 positive and negative groups, stratified by age into children (5 to 11 years) and youths (12 to 20 years).

	Children		Youths	
	COVID-19 positive cohort (N = 141,349)	COVID-19 Negative cohort (N = 441,790)	COVID-19 positive cohort (N = 184,725)	COVID-19 negative cohort (N = 445,524)
**Mean (SD) age (years)**	8·02 (2·03)	7·68 (2·02)	15·85 (2·48)	15·72 (2·46)
**Sex**				
female	67200(47·54%)	208647(47·23%)	102352(55·41%)	243311(54·61%)
male	74147(52·46%)	233128(52·77%)	82340(445·7%)	202124(45·37%)
other/unknown	2(0·00%)	15(0·00%)	33(0·02%)	89(0·02%)
**Race**				
Asian/PI	7147(5·06%)	22394(5·07%)	7103(3·85%)	19679(4·42%)
Black/AA	26028(18·41%)	77109(17·45%)	32121(17·39%)	71179(15·98%)
Hispanic	33470(23·68%)	99570(22·54%)	40774(22·07%)	87388(19·61%)
White	58446(41·35%)	190066(43·02%)	88475(47·90%)	223170(50·09%)
Multiple	2964(2·10%)	11100(2·51%)	2528(1·37%)	7739(1·74%)
other/unknown	13294(9·41%)	41551(9·41%)	13724(7·43%)	36369(8·16%)
**Hospital**				
A	10267(7·26%)	37518(8·49%)	12060(6·53%)	30577(6·86%)
B	17983(12·72%)	50147(11·35%)	17151(9·28%)	39349(8·83%)
C	5102(3·61%)	26782(6·06%)	5533(3·00%)	23306(5·23%)
D	4693(3·32%)	13587(3·08%)	6934(3·75%)	17011(3·82%)
E	4154(2·94%)	8044(1·82%)	7091(3·84%)	13052(2·93%)
F	2316(1·64%)	12643(2·86%)	2147(1·16%)	10349(2·32%)
G	1971(1·39%)	4023(0·91%)	4222(2·29%)	7944(1·78%)
H	3199(2·26%)	14347(3·25%)	8144(4·41%)	29957(6·72%)
I	1918(1·36%)	4976(1·13%)	5159(2·79%)	9732(2·18%)
J	2823(2·00%)	12582(2·85%)	3572(1·93%)	12225(2·74%)
K	2065(1·46%)	5921(1·34%)	2885(1·56%)	7978(1·79%)
L	13633(9·64%)	35395(8·01%)	12489(6·76%)	26245(5·89%)
M	7800(5·52%)	39868(9·02%)	9978(5·40%)	34533(7·75%)
N	449(0·32%)	1224(0·28%)	2084(1·13%)	3444(0·77%)
O	8372(5·92%)	32867(7·44%)	7897(4·28%)	24043(5·40%)
P	5565(3·94%)	15017(3·40%)	9188(4·97%)	22894(5·14%)
Q	2970(2·10%)	11581(2·62%)	4149(2·25%)	13249(2·97%)
R	20044(14·18%)	48775(11·04%)	28030(15·17%)	47454(10·65%)
S	7534(5·33%)	3709(0·84%)	11109(6·01%)	3666(0·82%)
T	1253(0·89%)	8849(2·00%)	1298(0·70%)	8494(1·91%)
U	3978(2·81%)	13696(3·10%)	4729(2·56%)	15025(3·37%)
V	3936(2·78%)	13460(3·05%)	4514(2·44%)	16603(3·73%)
W	4010(2·84%)	12124(2·74%)	6674(3·61%)	11213(2·52%)
X	3726(2·64%)	10243(2·32%)	5953(3·22%)	11553(2·59%)
Y	1588(1·12%)	4412(1·00%)	1735(0·94%)	5628(1·26%)
**BMI category**				
Non-obese	55731(39·43%)	208023(47·09%)	68050(36·84%)	203291(45·63%)
obese	72423(51·24%)	190405(43·10%)	96663(52·33%)	192282(43·16%)
Unknown	13195(9·34%)	43362(9·82%)	20012(10·83%)	49951(11·21%)
**Clinical characteristics**				
**ED visits**				
0	105627(74·73%)	328426(74·34%)	141487(76·59%)	342382(76·85%)
1	20116(14·23%)	67784(15·34%)	24140(13·07%)	62116(13·94%)
2+	15606(11·04%)	45580(10·32%)	19098(10·34%)	41026(9·21%)
**Inpatient visits**				
0	132890(94·02%)	413667(93·63%)	170628(92·37%)	405541(91·03%)
1	4998(3·54%)	19330(4·38%)	8518(4·61%)	26133(5·87%)
2+	3461(2·45%)	8793(1·99%)	5579(3·02%)	13850(3·11%)
**Outpatient visits**				
0	19623(13·88%)	72984(16·52%)	26547(14·37%)	72841(16·35%)
1	17936(12·69%)	71995(16·30%)	25188(13·64%)	70350(15·79%)
2+	103790(73·43%)	296811(67·18%)	132990(71·99%)	302333(67·86%)
**PMCA index**				
0	106350(75·24%)	326474(73·90%)	139008(75·25%)	322992(72·50%)
1	22402(15·85%)	71193(16·11%)	27216(14·73%)	71026(15·94%)
2	12597(8·91%)	44123(9·99%)	18501(10·02%)	51506(11·56%)
**Negative tests prior entry**				
0	84429(59·73%)	337684(76·44%)	121218(65·62%)	348636(78·25%)
1	31703(22·43%)	69760(15·79%)	36881(19·97%)	65033(14·60%)
2+	25217(17·84%)	34346(7·77%)	26626(14·41%)	31855(7·15%)

**Table 2:** Raw frequency of individual and composite neuropsychiatric and related conditions before and afterthe index date in the children cohort (5 to 11 years). For COVID-19 negative group, index dates areimputed randomly from the distribution of index dates observed in the COVID-19 positive cohort.

	COVID-19 Positive cohort		COVID-19 Negative cohort	
	Pre-COVID*	Post-COVID**	Pre-COVID	Post-COVID
**Any mental health disorder**	12·45%	14·01%	11·60%	12·48%
**Adverse Childhood Experiences**				
Emotional Abuse	0·04%	0·04%	0·02%	0·04%
Neglect	0·01%	0·02%	0·02%	0·01%
Physical Abuse	0·10%	0·10%	0·11%	0·10%
Sexual Abuse	0·05%	0·06%	0·06%	0·06%
**Anxiety Disorders**				
Anxiety Disorder	2·22%	2·93%	1·81%	2·23%
OCD	0·04%	0·19%	0·04%	0·12%
Somatoform Disorder	0·23%	0·31%	0·19%	0·23%
Stress Disorder	1·45%	1·73%	1·24%	1·42%
**Disruptive Behavior Disorders**				
Conduct Disorder	0·47%	0·46%	0·49%	0·52%
Impulse Control Disorder	0·40%	0·43%	0·43%	0·48%
Oppositional Defiant Disorder	0·30%	0·36%	0·30%	0·32%
**Eating and Feeding Disorders**				
Avoidant/Restrictive Food Intake	0·33%	0·39%	0·29%	0·32%
Other Eating and Feeding Disorder	0·09%	0·10%	0·08%	0·10%
**Elimination Disorders**				
Encopresis	0·14%	0·16%	0·21%	0·20%
Enuresis	0·64%	0·65%	0·62%	0·61%
**Gender Dysphoria/Sexual Dysfunction**				
Gender Dysphoria	0·04%	0·04%	0·04%	0·05%
Paraphilia	0·00%	0·00%	0·00%	0·00%
Sexual Dysfunction	0·00%	0·00%	0·00%	0·00%
**Intentional Self-Harm/Suicidality**				
Parasuicidality	0·07%	0·09%	0·07%	0·09%
Suicidality	0·14%	0·18%	0·13%	0·16%
**Mood Disorders**				
Bipolar Disorder	0·05%	0·07%	0·04%	0·05%
Major Depression	0·21%	0·27%	0·19%	0·25%
Minor Depression	0·31%	0·47%	0·27%	0·39%
**Neurocognitive Disorders**				
Catatonia	0·00%	0·00%	0·00%	0·00%
Delirium	0·09%	0·13%	0·09%	0·09%
Encephalopathy	0·04%	0·05%	0·05%	0·06%
**Neurodevelopmental Disorders**				
Academic Developmental Disorder	0·68%	0·77%	0·62%	0·72%
ADHD	4·25%	5·08%	3·58%	4·28%
Autism Spectrum Disorder	2·12%	2·32%	2·17%	2·29%
Communication/Motor Disorder	2·57%	2·41%	2·78%	2·53%
Intellectual Disability	1·20%	1·28%	1·34%	1·33%
**Personality Disorders**				
Personality Disorder	0·02%	0·02%	0·02%	0·02%
**Psychotic Disorders**				
Psychotic Disorder	0·02%	0·04%	0·03%	0·03%
Schizoaffective Disorder	0·00%	0·00%	0·00%	0·00%
Schizophrenia	0·00%	0·00%	0·00%	0·00%
**Sleep-Wake Disorders**				
Hypersomnia	0·06%	0·06%	0·06%	0·06%
Insomnia	0·47%	0·51%	0·46%	0·48%
Parasomnias	0·22%	0·25%	0·25%	0·24%
**Standalone Symptoms**				
Anger/Aggression	0·27%	0·32%	0·28%	0·32%
Anxiety Symptoms	0·28%	0·34%	0·25%	0·27%
Attention Symptoms	0·47%	0·56%	0·43%	0·49%
Depressive Symptoms	0·02%	0·02%	0·02%	0·02%
Hallucinations	0·13%	0·05%	0·09%	0·04%
**Substance Use and Dependence**				
Alcohol	0·00%	0·00%	0·00%	0·00%
Opioid Related	0·01%	0·01%	0·01%	0·00%
Other Substances	0·02%	0·02%	0·02%	0·01%
THC	0·00%	0·00%	0·00%	0·00%
Tobacco	0·00%	0·00%	0·00%	0·00%
**Tic Disorders**				
Tic Disorder	0·32%	0·37%	0·29%	0·30%

* Pre-COVID: Visit dates are between 24 months to 7 days before the index date

** Post-COVID: Visit dates are between 28–179 days after the index date

**Table 3:** Raw frequency of individual and composite neuropsychiatric and related conditions before and afterthe index date in the youths cohort (12 to 20 years). For COVID-19 negative group, index dates areimputed randomly from the distribution of index dates observed in the COVID-19 positive cohort.

	COVID-19 Positive cohort		COVID-19 Negative cohort	
	Pre-COVID*	Post-COVID**	Pre-COVID	Post-COVID
**Any mental health disorder**	16·00%	17·86%	15·55%	16·76%
**Adverse Childhood Experiences**				
Emotional Abuse	0·03%	0·03%	0·03%	0·02%
Neglect	0·01%	0·01%	0·02%	0·02%
Physical Abuse	0·13%	0·13%	0·16%	0·14%
Sexual Abuse	0·09%	0·10%	0·11%	0·10%
**Anxiety Disorders**				
Anxiety Disorder	6·88%	7·98%	6·19%	7·04%
OCD	0·10%	0·47%	0·12%	0·46%
Somatoform Disorder	0·49%	0·55%	0·43%	0·54%
Stress Disorder	2·60%	2·90%	2·33%	2·60%
**Disruptive Behavior Disorders**				
Conduct Disorder	0·24%	0·23%	0·26%	0·27%
Impulse Control Disorder	0·40%	0·42%	0·44%	0·46%
Oppositional Defiant Disorder	0·33%	0·33%	0·39%	0·36%
**Eating and Feeding Disorders**				
Avoidant/Restrictive Food Intake	0·90%	1·04%	0·97%	1·21%
Other Eating and Feeding Disorder	0·05%	0·06%	0·07%	0·07%
**Elimination Disorders**				
Encopresis	0·03%	0·03%	0·05%	0·04%
Enuresis	0·19%	0·18%	0·21%	0·18%
**Gender Dysphoria/Sexual Dysfunction**				
Gender Dysphoria	0·30%	0·36%	0·58%	0·64%
Paraphilia	0·01%	0·00%	0·01%	0·01%
Sexual Dysfunction	0·02%	0·02%	0·02%	0·02%
**Intentional Self-Harm/Suicidality**				
Parasuicidality	0·25%	0·30%	0·31%	0·35%
Suicidality	0·87%	0·99%	1·09%	1·13%
**Mood Disorders**				
Bipolar Disorder	0·16%	0·17%	0·15%	0·16%
Major Depression	3·55%	3·84%	3·58%	3·95%
Minor Depression	3·44%	4·25%	3·19%	3·76%
**Neurocognitive Disorders**				
Catatonia	0·02%	0·03%	0·02%	0·03%
Delirium	0·36%	0·48%	0·39%	0 42%
Encephalopathy	0·05%	0·07%	0·07%	0·08%
**Neurodevelopmental Disorders**				
Academic Developmental Disorder	0·41%	0·41%	0·46%	0·43%
ADHD	4·48%	4·79%	4·23%	4·30%
Autism Spectrum Disorder	1·22%	1·28%	1·50%	1·51%
Communication/Motor Disorder	0·51%	0·53%	0·65%	0·63%
Intellectual Disability	0·83%	0·87%	1·09%	1·06%
**Personality Disorders**				
Personality Disorder	0·13%	0·16%	0·13%	0·18%
**Psychotic Disorders**				
Psychotic Disorder	0·12%	0·15%	0·17%	0·20%
Schizoaffective Disorder	0·02%	0·03%	0·03%	0·04%
Schizophrenia	0·05%	0·07%	0·06%	0·08%
**Sleep-Wake Disorders**				
Hypersomnia	0·13%	0·15%	0·16%	0·16%
Insomnia	0·75%	0·90%	0·80%	0·84%
Parasomnias	0·12%	0·14%	0·17%	0·16%
**Standalone Symptoms**				
Anger/Aggression	0·28%	0·29%	0·35%	0·33%
Anxiety Symptoms	0·31%	0·38%	0·32%	0·32%
Attention Symptoms	0·35%	0·44%	0·33%	0·35%
Depressive Symptoms	0·04%	0·05%	0·04%	0·04%
Hallucinations	0·40%	0·11%	0·41%	0·12%
**Substance Use and Dependence**				
Alcohol	0·09%	0·10%	0·08%	0·09%
Opioid Related	0·03%	0·04%	0·03%	0·04%
Other Substances	0·14%	0·17%	0·16%	0·19%
THC	0·20%	0·25%	0·23%	0·28%
Tobacco	0·42%	0·51%	0·33%	0·41%
**Tic Disorders**				
Tic Disorder	0·28%	0·30%	0·32%	0·31%

* Pre-COVID: Visit dates are between 24 months to 7 days before the index date

** Post-COVID: Visit dates are between 28–179 days after the index date

## Data Availability

The regulatory documents, requests for data access, and other relevant study materials are available via the RECOVER website: https://recoverCOVID.org/
